# Normal Mode Splitting in a Moving-Particles-Pumped Mechanical Oscillator: Clamped-Hinged Homogeneous Beam

**DOI:** 10.1038/s41598-018-27989-8

**Published:** 2018-06-28

**Authors:** Zhi Sun

**Affiliations:** Research Fellow. State Key Laboratory for Disaster Reduction in Civil Engineering, Shanghai, 200092 China

## Abstract

The interaction of a mechanical oscillator with the operation actions and environment fields will give rise to the splitting of normal modes. In this study, we investigate the normal mode splitting behaviour of a moving-particles-pumped single-span clamped-hinged elastic homogeneous beam based on the proposed multi-octave modal parametric oscillation model. Numerical experiments show the entangled and squeezed oscillations of the phase-conjugated waves near the resonance tongues. Theoretical analysis predicts the occurrence of multiple simple resonances and the squeezing of the twin waves modal oscillation in the region with a low speed of movement for the studied system.

## Introduction

The interaction of a mechanical oscillator with the time-varying operation actions and environment fields may give rise to twin waves entanglement and squeezing behaviours. Attached moving articles are the most general operation actions and environmental disturbances for different forms of mechanical oscillators from the nano-scale devices to the large-scale infrastructures^1–4^. Considering the moving inertial effect of the attached particle, structural normal mode oscillations are parametrically modulated^5–8^. Although the parametric modulation gives rise to normal mode splitting and twin waves oscillation behaviours that have been observed in different physical systems^9–12^, to the best of our knowledge based on a survey of the literature, these behaviours have not been reported for the system studied in this investigation. It appears that the classical analysis of the studied system is generally based on an implicit assumption that these twin waves behaviours are found in a squeezed form or are short-lived. However, many numerical and experimental studies on this type of system^13–16^ have reported colourful eccentric oscillation behaviours. Recent analytical work^8,17^ has shown that the moving particle pumping effect can be modelled as multi-octave parametric excitation of the structure modal oscillations. In this study, we investigate the moving-particles-pumped normal mode splitting and twin waves characteristics of a single-span clamped-hinged elastic beam around the parametric resonance tongues based on this model. Numerical experiments based on the finite element method (FEM) structure modelling are conducted.

**Figure 1:**
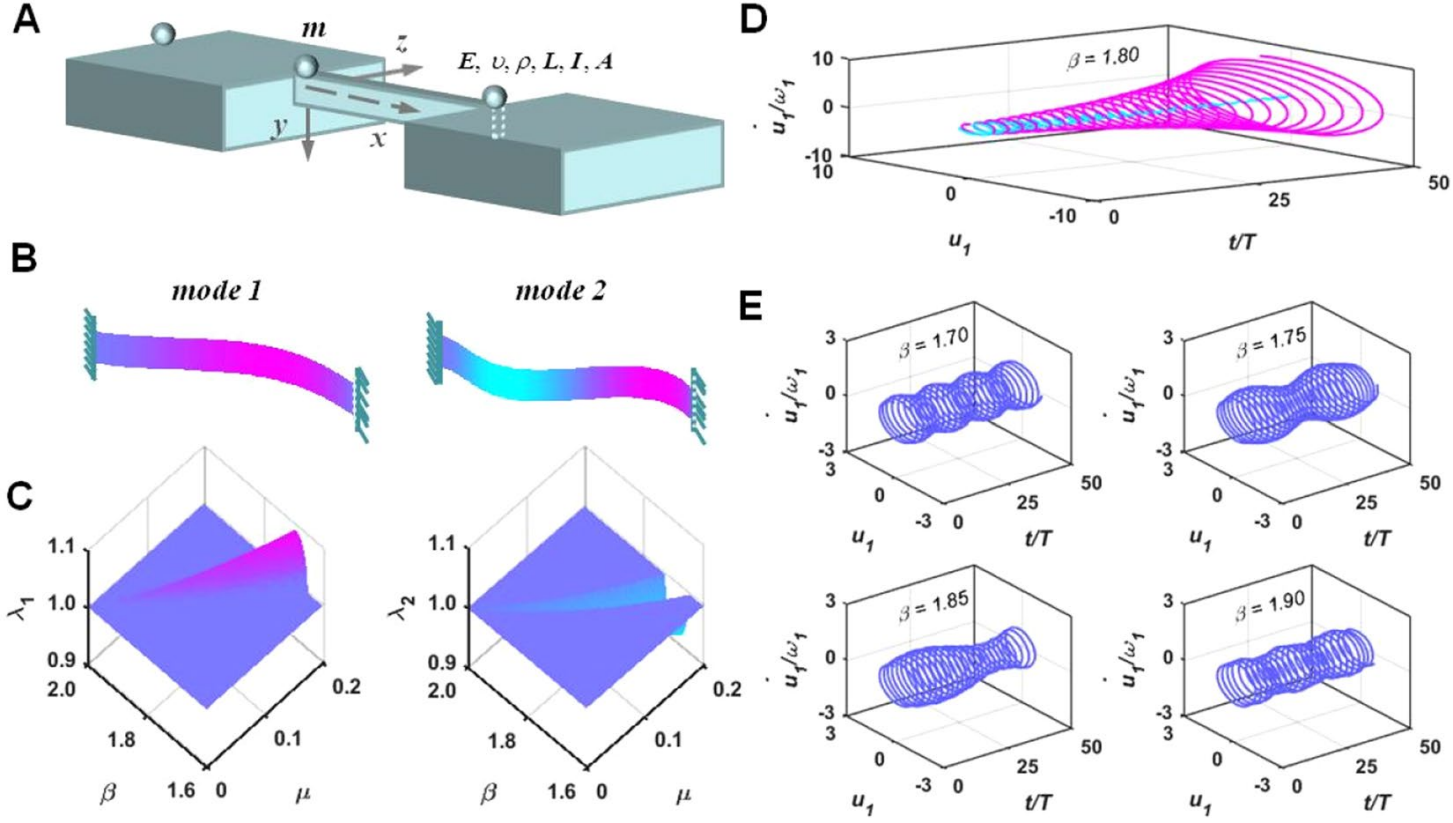
Tested structure and moving-particles-pumped amplitude modulation for the split fundamental mode around the principal resonance tongue. (**A**) Clamped-hinged beam-like structure transporting moving particle; (**B**) Mode shapes of the first two transverse flexural modes; (**C**) Tri-phase plots of the Floquet multiplier distribution on the *μ* − *β* plane for the studied case around the principal resonance tongues of the fundamental mode based on the MOMPO model analysis; (**D**) Numerically calculated amplitude-amplified-wave-dominated modal response phase trajectory (the magenta line) under *w*_1_(0) = 0, ${\dot{w}}_{1}$(0)/*ω*_1_ = 1 and amplitude-suppressed-wave-dominated modal response phase trajectory (the azure line) under *w*_1_(0) = 1, ${\dot{w}}_{1}$(0)/*ω*_1_ = 0 in the principal resonance of the fundamental mode when *μ* = 0.1 and *β* = 1.8 based on FEM structure modelling; (**E**) Numerically calculated modal response phase trajectories based on FEM structure modelling around the principal resonance tongue of the fundamental mode under initial disturbance of *w*_1_(0) = 1, ${\dot{w}}_{1}$(0)/*ω*_1_ = 1 when *μ* = 0.1.

The studied single-span structure is a clamped-hinged (CH) beam with uniform cross-section as shown in Fig. 1A. Its first two transverse vibration mode shapes are shown in Fig. 1B. Under a stream of moving particles with equal spacing distance, the governing equation of motion for the *z*-direction transverse vibration of the beam can be expressed as

1 $$EI\frac{{\partial }^{4}w(x,t)}{\partial {x}^{4}}+\rho A\frac{{\partial }^{2}w(x,t)}{\partial {t}^{2}}=-\,\sum _{i=1}^{{N}_{{\rm{o}}}}m{\ddot{q}}_{i}\delta (x-v{t}_{i})$$

where *ρA*, *EI*(*x*), *L*, and *w*(*x*, *t*) denote the mass per unit length, flexural rigidity, span length, and flexural deformation response of the supporting structure; *x* is the longitudinal coordinate of the supporting structure, with the origin at the left end-support of the structure; δ is the Dirac delta function; *m*, *q*_*i*_(*t*), *v*, and *t*_*i*_ = *t* − (*i* − 1)*T* denote the mass, transverse displacement, travelling speed, and travelling time, respectively, of the *i*-th particle on the supporting structure; $T=\frac{{l}_{{\rm{m}}}}{v}$; and *l*_m_ is the spacing distance of the moving particle stream. In this study, the cases when *l*_m_ = *L* are discussed. Considering the *z*-direction displacement compatibility at the instant of *t* for the *i*-th particle and the beam at the contact point of *x* = *vt*_*i*_

2 $${q}_{i}(t)=\{\begin{array}{ll}w(v{t}_{i},t) & (i-1)T\le t\le iT\\ 0 & {\rm{others}}\end{array}$$

When the particle is light compared to the supporting structure, its inertial perturbation to beam normal modes is small. Structural vibrations can still be efficiently described using the unperturbed orthogonal basis as

3 $$\begin{array}{c}(M+m{\varphi }_{r}{\varphi }_{r}){\ddot{w}}_{r}+{\rm{2}}m{\varphi }_{r}{\dot{\varphi }}_{r}{\dot{w}}_{r}+(M{\omega }_{r}^{2}+m{\varphi }_{r}{\ddot{\varphi }}_{r}){w}_{r}\\ \,\,\,\,\,\,\,+\sum _{s\ne r}m{\varphi }_{r}{\varphi }_{s}{\ddot{w}}_{s}+\sum _{s\ne r}{\rm{2}}m{\varphi }_{r}{\dot{\varphi }}_{s}{\dot{w}}_{s}+\sum _{s\ne r}m{\varphi }_{r}{\ddot{\varphi }}_{s}{w}_{s}={\rm{0}}\end{array}$$

where *w*_*r*_(*t*) is the response of the *r*-th structure modal coordinate; · denotes the differentiation with respect to time *t*; $M={\int }_{{\rm{0}}}^{L}\rho Adx$; *ω*_*r*_ are the natural frequencies of the *r*-th mode; and *φ*_*r*_ is the mode shape of the *r*-th mode satisfying ${\int }_{{\rm{0}}}^{L}\rho A{\varphi }_{r}^{2}(x)dx=M$. Since ${\varphi }_{r}{\varphi }_{s},{\varphi }_{r}{\dot{\varphi }}_{s},{\varphi }_{r}{\ddot{\varphi }}_{s}$ are continuous periodic functions with period *T* that can be expressed as harmonic series, this system is essentially a coupled multi-octave modal parametric oscillation (MOMPO) system. The multi-octave parametric excitation will pump multiple parametric resonance tongues around *ω* ≈ 2*ω*_*r*_/*n* (*r*, *n* ∈ *Z*) along the frequency axis where *ω* = 2*πv*/*L*.

Focusing on the principal resonance tongue of the fundamental mode, the principal components of the modal oscillation around the transition curves are analysed according to the Floquet theory^18^ and are composed of twin modulated harmonic waves

4 $${w}_{1}(t)={A}_{1}{e}^{{\gamma }_{1}\omega t}\,\sin ({\Omega }_{1}\omega t+{\theta }_{1})+{A}_{2}{e}^{{\gamma }_{2}\omega t}\,\sin ({\Omega }_{2}\omega t+{\theta }_{2})$$

where *γ*_1_, *γ*_2_ and *Ω*_1_*ω*, *Ω*_2_*ω* are the amplitude modulation characteristics of exponentials and modulated frequencies; *A*_1_, *A*_2_ and *θ*_1_, *θ*_2_ are the amplitudes and phase angles related to the given initial conditions as well as system characteristics. The determination of *γ*_1_, *γ*_2_ and *ωΩ*_1_, *ωΩ*_2_ is based on the numerical computation of the one-period response of the system to the unit initial displacement and velocity disturbances. If the computed one-period displacement response to the unit initial displacement disturbance is denoted as *w*_1,1_(*T*) and the computed one-period velocity response to the unit initial velocity disturbance is denoted as ${\dot{w}}_{1,2}$(*T*), then ${\gamma }_{1},\,{\gamma }_{2}=\mathrm{Re}(\mathrm{ln}(\alpha \pm \sqrt{{\alpha }^{2}-1})/2\pi )$, ${\Omega }_{1}=\text{Im}(\mathrm{ln}(\alpha +\sqrt{{\alpha }^{2}-1})/2\pi )$, and ${\Omega }_{2}=1+\text{Im}(\mathrm{ln}(\alpha -\sqrt{{\alpha }^{2}-1})/2\pi )$, where $\alpha =[{w}_{1,1}(T)+{\dot{w}}_{1,2}(T)]/2$.

For the system described by Eq. (1), the principal component around the resonance tongues is composed of twin modulated harmonic waves. Figure 1C presents the tri-phase and contour plots of the computed characteristics multiplier (*λ*_1_, *λ*_2_) distributions on the *μ* − *β* plane (*μ* = *m*/*M*, *β* = *ω*/*ω*_1_) for the twin waves of the fundamental mode around the principal resonance tongue. It can be seen that around this resonance tongue heading to *β* = 2.0 with an oblique angle, the amplitude modulation characteristics of exponentials (*γ*_1_, *γ*_2_) for the twin waves satisfy the conditions *γ*_1_ ≈ −*γ*_2_ for the in-resonance region and *γ*_1_ = *γ*_2_ for the out-of-resonance region. For *μ* = 0.1 and *β* = 1.8, which is found in the principal resonance tongue of the fundamental mode, the computed phase trajectories based on the FEM structure modelling (as shown in Fig. 1D) considering a stream of 50 particles passing the beam verify that the amplitude-amplified wave dominates the modal oscillation when *w*_1_(0) = 0, ${\dot{w}}_{1}$(0)/*ω*_1_ = 1 and the amplitude-suppressed wave dominates the oscillation when *w*_1_(0) = 1, ${\dot{w}}_{1}$(0)/*ω*_1_ = 0. To further check the oscillations near the transition curve out of the resonance tongues for *μ* = 0.1 and *β* = 1.70, 1.75, 1.85, 1.90, Fig. 1E presents the computed oscillation phase trajectories for the fundamental mode. It can be seen that in this degenerate characteristic exponential region, the twin modulated harmonics oscillation with close frequencies and similar amplitudes presents beats shape displacement and velocity oscillations.

**Figure 2:**
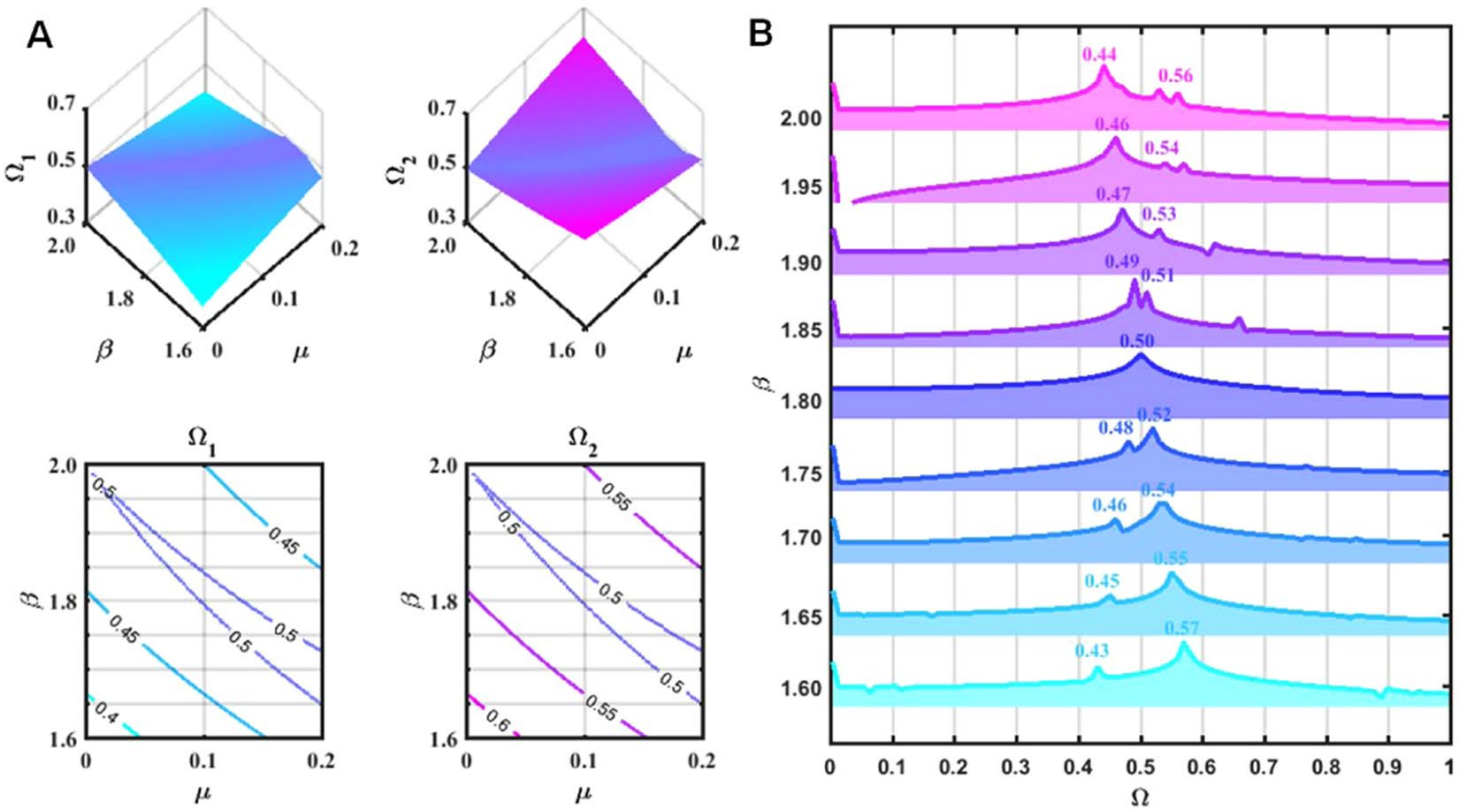
Moving-particles-pumped fundamental mode spectrum splitting of the CH beam near the principal resonance tongue. (**A**) Triphase and contour plots of the predicted frequency modulation characteristics distribution on the *μ* − *β* plane for clamped-hinged uniform beams carrying moving particles around the principal resonance of the fundamental mode based on MOMPO model analysis; (**B**) Numerically calculated amplitude spectrum based on FEM structure modelling for the fundamental mode response oscillation under a stream of 100 particles around the principal resonance tongue with initial disturbance of *w*_1_(0) = 1, ${\dot{w}}_{1}$(0)/*ω*_1_ = 1 when *μ* = 0.1.

The results obtained in a detailed investigation of the frequency modulation characteristics around the principal resonance tongue for *n* = 1 are shown in Fig. 2. Figure 2A presents the tri-phase and the contour plots of *Ω*_1_ and *Ω*_2_ for the fundamental mode based on the MOMPO model analysis. It can be seen that *Ω*_1_ = *Ω*_2_ = *n*/2 for the in-resonance region and *n*/2 − *Ω*_1_ = *Ω*_2_ − *n*/2 for the out-of-resonance region. FEM structure modelling based calculated amplitude spectra of the fundamental modal displacement responses are presented in Fig. 2B. It can be seen that for the in-resonance region (*μ* = 0.1, *β* = 1.8), the two components show the same oscillation frequency and present a single peak at *Ω* = 0.50 in the amplitude spectrum. For the out of-the-resonance region, the oscillation amplitude spectrum peaks for the twin waves are symmetrically distributed with respect to the axis of *Ω* = 0.50. When *β* approaches 2.0, little variation is observed among the *Ω*_1_s and *Ω*_2_s computed from the MOMPO model considering the fundamental mode and from the FEM structure modelling-based numerical experiments. Considering that the 1/2 sum combination resonance tongue for modes 1 and 2 heading to *β* = 2.13 is placed nearby, the observed deviations are reasonable. Figure 2B also shows that among the twin waves, the modulated harmonics with the singularity red-shifted frequency in the region above the resonance tongue and the modulated harmonics with singularity blue-shifted frequency in the region below the resonance tongue dominate the modal oscillation when *ω* is away from the resonance tongues.

**Figure 3:**
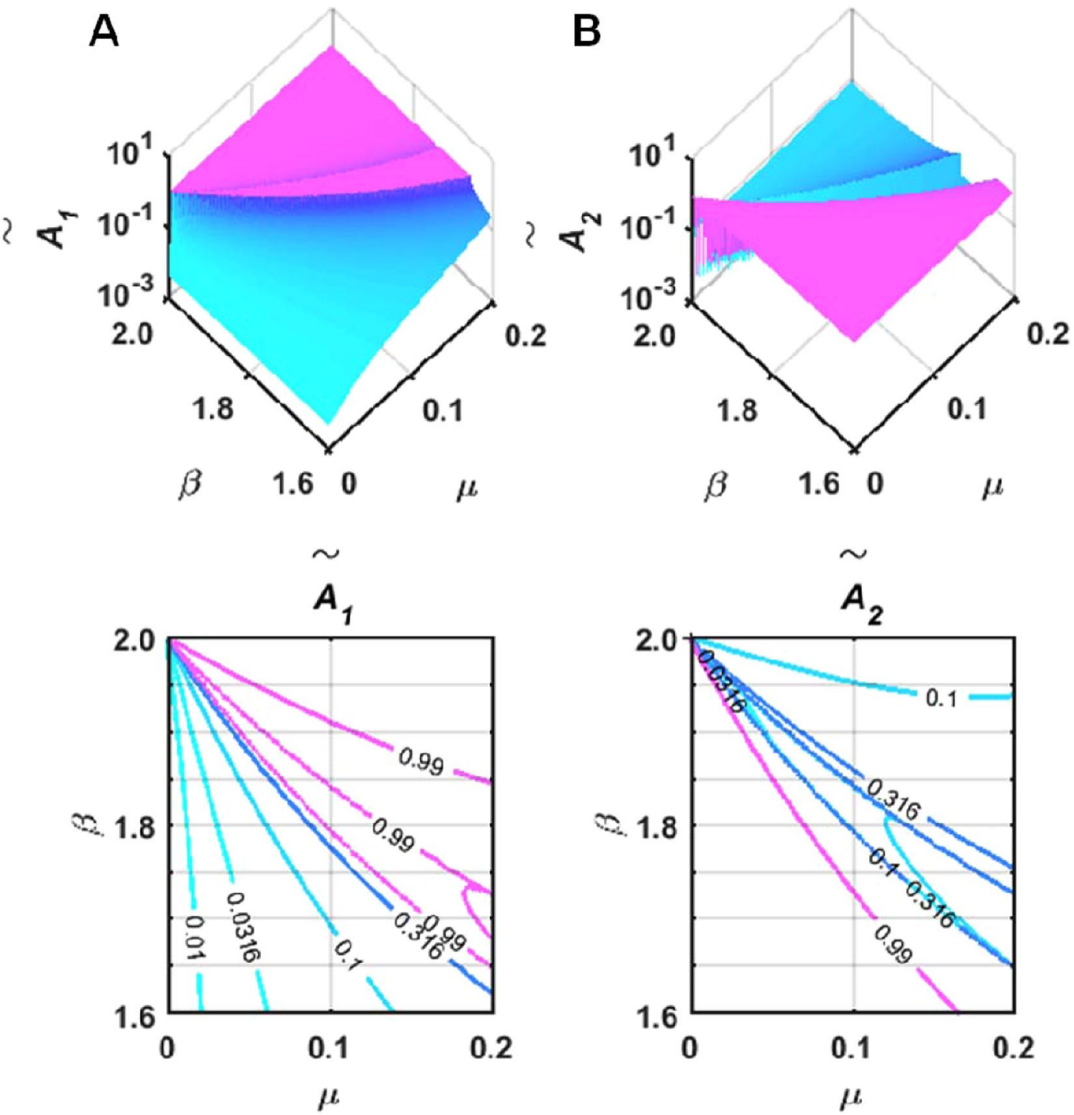
Moving-particles-pumped twin waves normalized amplitudes around the principal resonance tongue for the fundamental mode of the CH beam based on the MOMPO model analysis. (**A**) Tri-phase and contour plots of the normalized amplitude of the singularity red-shifted components on the *μ* − *β* plane; (**B**) Tri-phase and contour plots of the normalized amplitude of the singularity blue-shifted components on the *μ* − *β* plane.

The variation trends of the twin-wave oscillation amplitudes on the *μ* − *β* plane are analysed according to the MOMPO model described by Eq. (1). Figure 3 presents the normalized oscillation amplitudes (${\tilde{A}}_{1}$, ${\tilde{A}}_{2}$) of the twin waves of the fundamental mode where ${\tilde{A}}_{1}=|{A}_{1}|/\sqrt{{A}_{1}^{2}+{A}_{2}^{2}}$ and ${\tilde{A}}_{2}=|{A}_{2}|/\sqrt{{A}_{1}^{2}+{A}_{2}^{2}}$, *A*_1_, *A*_2_ are the computed twin waves amplitudes. Figure 3A presents the tri-phase and contour plots of ${\tilde{A}}_{1}$ distribution on the *μ*−*β* plane. As shown, in the region below the resonance tongue, the normalized amplitude of the modulated harmonics with singularity red-shifted frequency quickly decreases from 0.99 to 0.01 along with the increase in the distance away from the resonance tongue. In the region above the resonance tongue, the plateau-shaped distribution around ${\tilde{A}}_{1}$ ≈ 0.99 demonstrates the dominance of this component in this region. Similarly, the ${\tilde{A}}_{2}$ distribution shown in Fig. 3B indicates that the modulated harmonics with the singularity blue-shifted frequency are vital in the region below the resonance tongue and are squeezed after going through the resonance tongue. The computed amplitude distribution surfaces determine the range where the modal oscillation can be approximated by single modulated harmonics.

**Figure 4:**
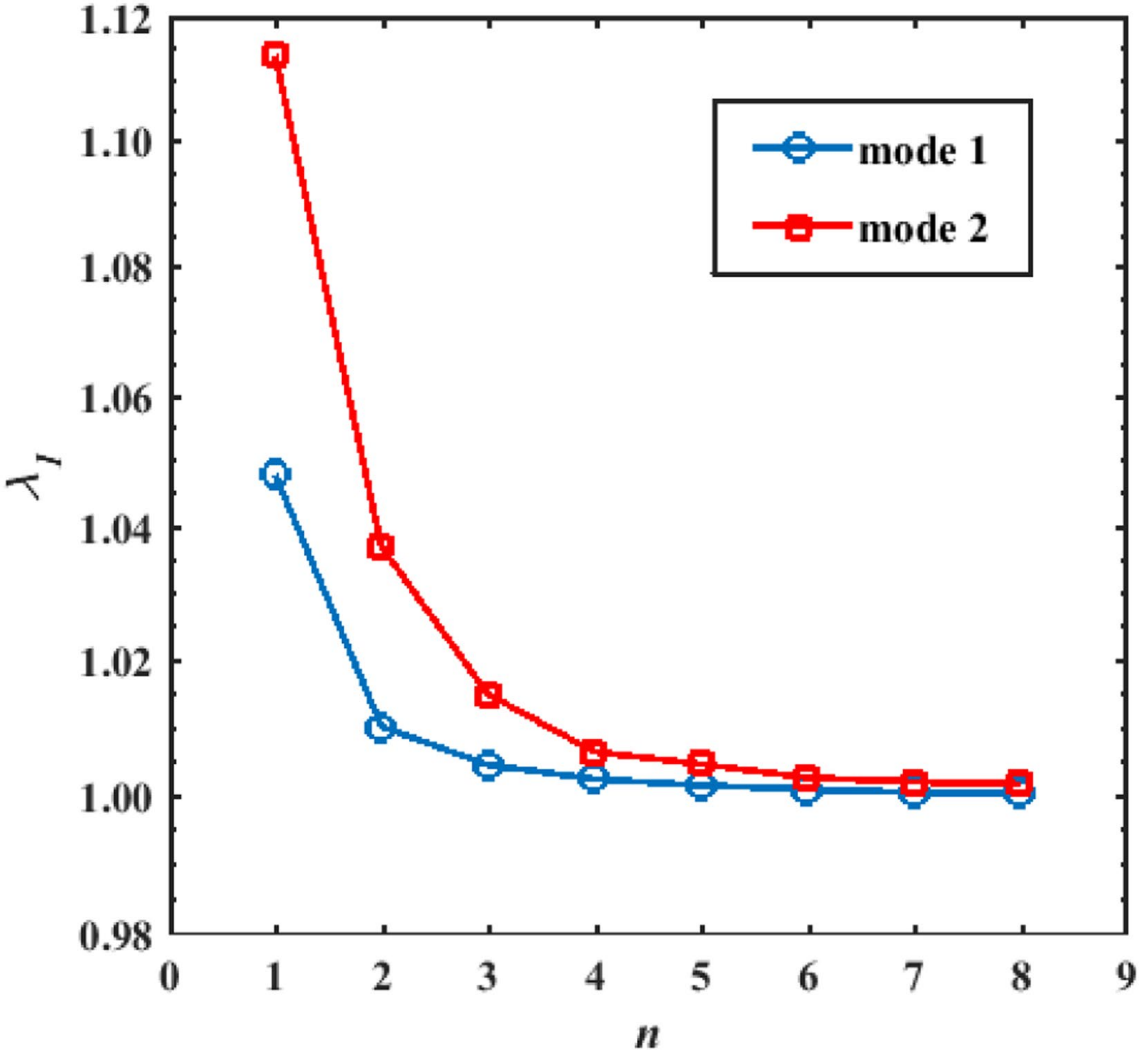
Moving-particles-pumped characteristics multiplier peak values for the simple resonance tongues of the first two modes of the studied structure based on the MOMPO model analysis when *μ* = 0.1.

In addition to the principal resonance, the higher-order simple parametric resonances will also induce twin waves oscillation behaviours. Since these resonances are predicted to occur in the regions with low moving speed of the same scale of importance, the related parametric amplification and twin waves behaviours must be investigated. Fig. 4 presents the computed peak characteristics multipliers *λ*_1_ for the simple resonance tongues of the first two modes of the CH beams based on the multi-octave modal parametric oscillation model when *μ* = 0.1. It can be seen that with the increase in *n*, the computed *λ*_1_ s monotonically decreased to 1. Moreover, this figure also shows that the peak characteristic multipliers of the second mode are larger than those of the fundamental mode. If the resonances of the higher-order modes are excited either owing to the described auto-mode parametric modulation or owing to the cross-mode coherent coupling, important parametric amplification may occur.

In this study, we investigate the moving-particles-pumped structure normal mode splitting and twin waves oscillation for the single-span CH homogeneous beam structures. The analytical modelling of the system as a multi-octave parametric excited system predicts the occurrence of the parametric resonance when *ω* ≈ 2*ω*_*r*_/*n* and the twin waves oscillation characteristics around the resonance tongues. FEM-based numerical experiments show the in-resonance amplitude-modulated and out-of-resonance frequency-modulated twin waves oscillation characteristics around the principal parametric resonance tongue for the fundamental mode. Away from the parametric resonance tongues, the twin waves will be squeezed to show the classical single modulated harmonics modal oscillation. If multiple structural modes considering geometric configuration, higher dimension effects, supporting conditions, particle moving routes, and environmental disturbance patterns are important, the mode splitting and multi-wave oscillation phenomena with varying patterns around the simple and combination resonance tongues will be ubiquitous and need to be carefully discriminated.

## Methods Summary

The analysis of twin waves oscillation for the system described by Eq. (1) is conducted based on the Floquet theory^18^. The tested CH beam-like structure was designed to be composed of steel with Young’s modulus *E* = 210 GPa, Poisson ratio *υ* = 0.3, and mass density *ρ* = 7800 *kg*/*m*^3^. The cross-section is a uniform rectangle along the *x-*axis with a length-height ratio *L*/*h* = 10 and a height-width ratio *h*/*b* = 10. The eigen-frequencies and mode shapes computation were conducted on an FEM model using the 12-DOFs Kirchhoff thin-plate element. Since the most important first three modes are the transverse flexural modes for this slender elastic continuum, the response computation was conducted on an FEM model using the 4-DOFs Euler/Bernoulli beam element, with a cross-section area of *A* = *bh* and a moment of inertia about the y-axis *I* = *b*^3^*h*/12, based on the Newmark method. Particle-structure interaction was simulated by updating the elemental stiffness, mass and damping matrices according to the particle mass and the shape function value at the particle on-element location.

### Data availability

The datasets generated and analysed in the FEM numerical experiments are available from the corresponding author on reasonable request.
